# O1.1 ALTERED COMPLEMENT PATHWAY PROTEIN EXPRESSION IS ASSOCIATED WITH PSYCHOTIC EXPERIENCES AT AGE 11 WHICH PERSIST AT AGE 18

**DOI:** 10.1093/schbul/sby015.183

**Published:** 2018-04-01

**Authors:** David Cotter, Jane English, Melanie Foecking, Mary Cannon, Bart Rutten, Stanley Zammit, Glyn Lewis, Sophie Sabhwerwal, Lorna Lopez, Aoife O’Gorman, Gerard Cagney

**Affiliations:** 1 Royal College of Surgeons in Ireland; 2 Royal College of Surgeons in Ireland, Beaumont Hospital; 3 Maastricht University Medical Centre; 4 Cardiff University; 5 University College London; 6 Proteome Research Centre, University College Dublin

## Abstract

**Background:**

The identification of early biomarkers of psychotic disorder is important because early treatment is associated with improved outcome. We have previously shown that altered complement and coagulation pathway associated proteins are associated pathway with psychotic disorder at age 18. In the current study we test the hypothesis that altered complement pathway proteins are associated with persisting psychotic experiences from age 11 to age 18.

**Methods:**

The Avon Longitudinal Study of Parents and Children (ALSPAC) is a prospective general population cohort, and a rich resource of demographic, environmental, and clinical data on the individuals involved. We studied a subsample of the cohort who participated in psychiatric assessment interviews at age 11 and 18, and who provided plasma samples at age 11. Semi-targeted proteomic profiling was used to specifically assess the complement pathway proteins in age 11 children who experienced psychotic experiences (but not disorder) at age 11 and age 18 (n=39) compared to age 11 children who only experienced psychotic experiences at age 11.

**Results:**

11 of 34 proteins assessed were significantly differentially expressed at p<0.05 and of these 8 remained significant following correction for multiple comparisons. Protein changes were in keeping with increased proteins expression of most complement pathway proteins. Several protein changes represented specific replications of the changes observed in age 11 samples prior to psychotic disorder at age 18, namely increased plasminogen, complement factor H, and complement factor 1r.

**Discussion:**

Our findings implicate the blood complement system in the persistence of psychotic experiences from age 11 to age 18. Considering that psychotic experiences are predictive of many psychiatric disorders our findings implicate the complement system not just in psychotic disorders, but more broadly in the vulnerability to a range of adult psychiatric disorders.

